# Managing Sleep and Behavioral Problems in a Preschooler with *SATB2*-Associated Syndrome

**DOI:** 10.1155/2020/8868458

**Published:** 2020-07-12

**Authors:** Nihit Kumar, Yuri A. Zarate

**Affiliations:** ^1^Division of Child & Adolescent Psychiatry, University of Arkansas for Medical Sciences, 4301 W. Markham, Slot 654, Little Rock, AR 72205, USA; ^2^Section of Genetics and Metabolism, University of Arkansas for Medical Sciences, Little Rock, AR, USA

## Abstract

*SATB2*-associated syndrome is an autosomal dominant, multisystemic disorder with associated sleep and behavioral abnormalities. Evidence is limited on appropriate management strategies in this population. We describe the medical management of a four-year-old child with poor sleep and significant behavioral problems. After failing initial treatment with melatonin, we initiated treatment clonidine along with high doses of trazodone for sleep. Daytime treatment with quetiapine was added to successfully manage behavioral issues. We present the challenges associated with treatment strategies in children with this syndrome.

## 1. Introduction

*SATB2*-associated syndrome (SAS) is a multisystemic disorder transmitted in an autosomal dominant pattern, with onset of symptoms before 2 years of age. It is associated with abnormalities of speech, palate, and teeth, with or without bone or brain anomalies, and behavioral issues [1, 2]. A recent study characterized the phenotype of children with SAS and reported that they have a high frequency of emotional problems, peer problems, and hyperactivity, with nearly half the individuals in this study having at least one sleep disorder [3]. However, there is a dearth of literature on the management of sleep and behavioral problems in these individuals. We present a case of a four-year-old with SAS and chronic sleep and behavioral problems who was treated with high doses of trazodone in combination with quetiapine and clonidine.

## 2. Case Report

The four-year-old Caucasian male was first evaluated by genetics at 15 months of age given his history of global developmental delay. On examination, he was noted to have hypotonia and with craniofacial dysmorphism that included a thin vermillion of the upper lip, a broad nasal bridge, and deeply set eyes but with normal palatal morphology. By 16 months of age, he underwent gastrostomy tube placement for severe dysphagia. Given the persistence of seizure-like spells with electroencephalograms (EEG) that revealed epileptiform discharges, he was started on levetiracetam at 3.5 years of age. This medication was weaned off at 5 years of age given the lack of clinical correlation between spells and EEG data. Over time, patient developed taurodontism and maloclussion, while bone density scan documented osteopenia at 4 years of age. Extensive evaluation for potential underlying abnormalities included brain and spinal imaging, the SNP chromosomal microarray analysis, spinal muscular atrophy molecular testing, SNRPN methylation, and fragile X testing, all within normal limits. The clinical exome sequencing analysis of 4616 genes revealed a *de novo* heterozygous pathogenic variant in *SATB2* (NM_015265.3 : c.1285C > T (p.R429∗)).

**Figure 1:**
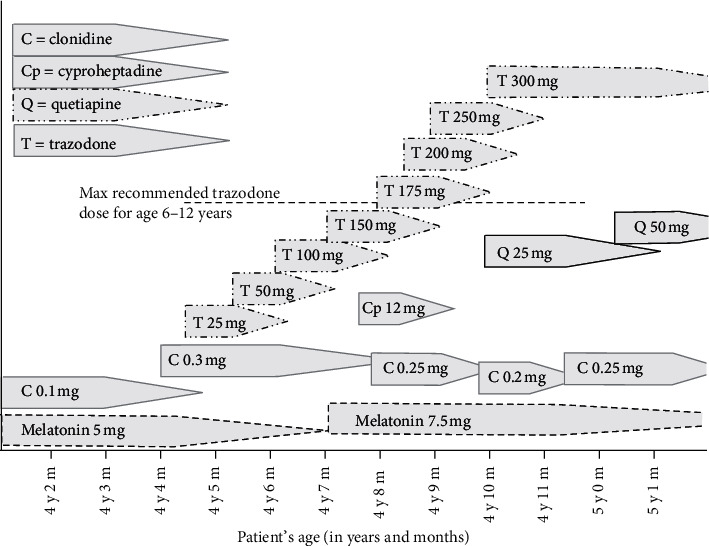
Schematic representation depicting the use of sleep and behavioral medications in a preschooler with *SATB2*-associated syndrome.

The child presented for initial evaluation in our outpatient child and adolescent psychiatry clinic for ongoing sleep and behavioral problems. Since the patient was nonverbal, the history was obtained primarily from his parents. During the initial evaluation, his parents reported that his sleep problems started around the age 2.5 years, with chronic maintenance insomnia. He would initiate sleep between 11 and 11:30 pm but would wake up around 4 am most mornings and would stay awake. He would sometimes take a nap for about an hour, either around 8-9 am or, once he started school, between 12 and 1 pm. There was no evidence of other kinds of sleep problems, including parasomnias, sleep-breathing disorders, restless leg syndrome, or sleep eating. His behavioral problems included climbing on parents lap and upper torso, claw/scratch their face or limbs, pull family members' hair, flap/clap his hands when excited, climb up on furniture, knock over objects, and kick and scream, especially when he would get bored or frustrated. He was unable to accurately assess his environment and was unaware of dangerous situations; for example, he would walk on to the street or walk into a pool. His medications at the time of initial presentation included levetiracetam 250 mg twice daily, clonidine 0.1 mg at bedtime, and melatonin 5 mg at around 8 pm (Figure 1). He was also on over the counter calcium and vitamin D (400 IU daily) supplements, iron supplements, fish oil (omega-3), bone broth, sage (*Salvia officinalis*), and cannabidiol (CBD) oil off and on. He received all his medications and supplements through his G-tube. A multispecialty team evaluation at around age 3 determined that he did not have autism spectrum disorder (ASD). He could not participate in the behavioral therapy due to not having access to specialized therapists who could work with nonverbal children. He did receive speech, occupational, and physical therapy and was working on learning a picture-based communication system. He also had a history of previous tonsillectomy and pressure equalization (PE) tubes. He did not have a history of a mental health disorder or abuse/maltreatment/neglect but did have a family history positive for anxiety and depression.

Given his continued sleep and behavioral problems, his clonidine was gradually increased from 0.1 mg at bedtime to 0.1 mg three times daily, and he was eventually started on trazodone, which was gradually increased to 75 mg at bedtime. Appropriate sleep hygiene education was given to parents, which they implemented. At this time, he averaged about 5-6 hours of nighttime sleep with 1.5–3 hour nap during the afternoons, but there were some nights that he only slept an hour. His behaviors improved some with less climbing and clawing at them; however, he started to “whine” a bit more and hit himself with his fists. Since he appeared a little stable with his sleep, parents tried to get him to sleep in his own toddler bed, which was attached to their bed; however, this did not sustain. An overnight polysomnogram at 4 years of age ruled out central or obstructive episodes of sleep apnea but showed reduced sleep efficiency (82%, normal >90%) and increased arousal index of 14.9 per hour (normal <10/hour). He was also started on ferrous sulfate 5.5 mL twice daily due to low ferritin levels (49.8 ng/mL). Since he continued to wake up around 4–4:30 am, we tried splitting his total dose of trazodone, which did not work as well, so his dose was consolidated to trazodone 150 mg at 10 pm. His neurologist also started him on cyproheptadine 4 mg three times daily for headaches, but this was discontinued a month later due to lack of effectiveness. Patient started prekindergarten at his new school by this time and was falling asleep in the mornings, so we decided to decrease the daytime dose of clonidine to 0.05 mg every morning and noon, while continuing with the bedtime dose at 0.1 mg. Trazodone was further increased in a step-wise manner to 300 mg given at 10 pm. He also started having daytime episodes of screaming for hours at a time, which parents described “as if he was hurting,” without any apparent reason. He was started on quetiapine 25 mg as needed for these screaming/agitated episodes, which was initially occurring once every other week. However, these worsened in frequency to daily episodes, and since quetiapine appeared to calm him down, he started receiving quetiapine 25 mg daily at around 4 pm, which was further increased to 50 mg due to lack of effect from the previous dose. He is G-tube fed, so parents control his caloric intake given the increased risk of metabolic syndrome due to weight gain with quetiapine. To monitor for side effects, his height and weight was regularly monitored during clinic visits. Periodic lab work including sodium, calcium, and phosphorous levels, vitamin D levels, hepatic and renal function, and glucose have been within normal ranges.

His current behavioral and sleep medications include clonidine 0.05 mg at breakfast, 0.1 mg at 2:30 pm, and 0.1 mg at 8pm, quetiapine 50 mg at 4 pm, melatonin 7.5 mg at 8 pm, and trazodone 300 mg at 10 pm. The maximum recommended pediatric dose for trazodone in children of age 6–12 years is 6 mg/kg/day, whereas we are using around 11.4 mg/kg/day in this four-year-old. Overall, his behaviors are much better, and minimal agitation was reported, and he is sleeping from about 9–9:30 pm until 4-5 am.

## 3. Discussion

*SATB2*-associated syndrome is a multisystemic disorder clinically characterized by significant neurodevelopmental compromise with absent or limited speech development. Dental abnormalities, feeding difficulties, suggestive facial features, palatal anomalies, skeletal anomalies (osteopenia, pectus deformities, kyphosis/lordosis, and scoliosis), and growth restriction complete the characteristic phenotype of SAS [2]. From a behavioral perspective, some commonly reported findings include autistic features, hyperactivity, aggressiveness, and significant sleeping difficulties [3].

This case highlights the potential challenges in managing sleep and behavioral problems in children with *SATB2*-associated syndrome. Nonpharmacologic interventions for insomnia including sleep hygiene education and the cognitive-behavioral therapy for insomnia (CBT-I) are generally effective [4, 5], but may not be in an option for children with SAS who are nonverbal and/or with lower IQ or residing in geographical areas with limited availability of trained specialists. Implementing therapies for behavior management may also be challenging for some of these same reasons. Another dilemma in children with SAS is to tease out the etiology of behaviors, as underlying mood, anxiety, pain, gastrointestinal disturbances (e.g., constipation), or sensory issues, could all cause behavioral problems. Also, in the United States, a lot of behavior management therapies are not covered by health insurance, especially in children who have not been formally diagnosed with ASD. Pharmacologic management in such situations emerges as a viable alternative option.

Even so, the evidence is limited on pharmacologic management of sleep disorders in children with neurodevelopmental disabilities [4]. As described in the case above, the typical weight-based dosing of trazodone for sleep was not very effective. Hence, we used high doses of trazodone, which was started at a lower dose and increased slowly and carefully. We monitored closely for hyponatremia, especially in this patient with co-occurring seizures and elevated liver function (AST, aspartate aminotransferase and ALT, alanine aminotransferase), which are some of the listed adverse effects of trazodone at high doses. We were careful not to use other serotonergic agents to avoid the possibility of serotonin syndrome. There are two US FDA (United States Food and Drug Administration) approved medications for treating irritability in children with ASD—aripiprazole and risperidone [6]. Our rationale for choosing quetiapine was its sleep-inducing effects and low metabolic risk given that parents controlled his caloric intake.

To our knowledge, this is the first time treatment challenges including the use of high-dose trazodone in managing sleep in a young child with *SATB2*-associated syndrome have been described. Some of the limitations include confounding effects by the use of concurrent medications, as well as not using an objective measure to track daily sleep data.

In conclusion, sleep and behavior problems are ubiquitous in children with *SATB2*-associated syndrome. Evidence-guiding appropriate management strategies are limited, and treatment can be challenging for a variety of reasons. Use of evidence-based nonpharmacological options should be first-line whenever possible, but practitioners should not shy away from using higher than recommended pharmacological doses with careful monitoring of risks and discussion with the patients' caregivers.
